# Transgelin-expressing myofibroblasts orchestrate ventral midline closure through TGFβ signalling

**DOI:** 10.1242/dev.152843

**Published:** 2017-09-15

**Authors:** Bashar Aldeiri, Urmas Roostalu, Alessandra Albertini, Jason Wong, Antonino Morabito, Giulio Cossu

**Affiliations:** 1 Manchester Academic Health Science Centre, Division of Cell Matrix Biology and Regenerative Medicine, School of Biological Sciences, Faculty of Biology, Medicine and Health, University of Manchester, Manchester M13 9PL, UK; 2 Royal Manchester Children's Hospital, Manchester M13 9WL, UK; 3 University Hospitals of South Manchester, Manchester M23 9LT, UK

**Keywords:** TGFβ, Transgelin, Myofibroblast, Midline defect, Exomphalos, Mouse

## Abstract

Ventral body wall (VBW) defects are among the most common congenital malformations, yet their embryonic origin and underlying molecular mechanisms remain poorly characterised. Transforming growth factor beta (TGFβ) signalling is essential for VBW closure, but the responding cells are not known. Here, we identify in mouse a population of migratory myofibroblasts at the leading edge of the closing VBW that express the actin-binding protein transgelin (TAGLN) and TGFβ receptor (TGFβR). These cells respond to a temporally regulated TGFβ2 gradient originating from the epithelium of the primary body wall. Targeted elimination of TGFβR2 in TAGLN^+^ cells impairs midline closure and prevents the correct subsequent patterning of the musculature and skeletal components. Remarkably, deletion of *Tgfbr2* in myogenic or chondrogenic progenitor cells does not manifest in midline defects. Our results indicate a pivotal significance of VBW myofibroblasts in orchestrating ventral midline closure by mediating the response to the TGFβ gradient. Altogether, our data enable us to distinguish highly regulated epithelial-mesenchymal signalling and successive cellular migration events in VBW closure that explain early morphological changes underlying the development of congenital VBW defects.

## Abstract (precis)

**Summary:** A population of migratory myofibroblasts at the leading edge of the closing ventral body wall expresses cytoskeletal components and TGFβR2 and responds to an epithelial TGFβ2 morphogen gradient to drive midline closure.

## INTRODUCTION

Abdominal wall defects are common in humans and cause significant morbidity and mortality (Wilson and Johnson, 2004). They show a variety of phenotypic abnormalities that differ not only in their anatomy, but also in their mode of development, organ involvement and short- and long-term outcomes (Carnaghan et al., 2013; Christison-Lagay et al., 2011; Gamba and Midrio, 2014; Sadler, 2010; Sadler and Feldkamp, 2008). Little is known about the mechanisms that drive ventral midline closure in mammals. The ventral body wall (VBW) in mice arises as a result of a turning process that transforms the ʻcup-shaped' embryo proper. During this morphogenetic process the initial ectodermal-mesodermal layer, known as the primary body wall, provides an initial thin cover to the embryonic endoderm at ∼E8.5-E9.5 (Kaufman, 1992). Starting from ∼E12, differentiated secondary mesenchymal components arising laterally from the flanks follow the primary body wall. This later cellular migration process continues through late embryonic stages, and complete migration and fusion of the secondary body wall elements is fully achieved by E15.5 in the thorax and E16.5 in the abdomen (Kaufman and Bard, 1999). The mechanisms that drive these large-scale morphogenetic movements and the cell types that are involved remain largely unknown. Similarly, a possible role of the cells of the primary body wall in facilitating ventral midline closure remains to be investigated. In diaphragm development, fibroblasts pilot the way for muscle cell migration and it is defects in these connective tissue fibroblasts that result in the development of congenital diaphragmatic hernia, rather than any primary muscle cell defect (Merrell et al., 2015). In addition, recent evidence suggests a role of epithelial-mesenchymal signalling not only in epithelium patterning, but also as a major regulator of secondary body wall element migration (Brewer and Williams, 2004; Budnick et al., 2016; Candille et al., 2004; Eng et al., 2012; Zhang et al., 2014). Nonetheless, the cellular and morphogenetic components of the epithelial-mesenchymal pathway in ventral midline closure remain largely obscure.

Transforming growth factor β (TGFβ) signalling was proven to play a pivotal role in facilitating closure of the midline in various tissues and body districts (Dünker and Krieglstein, 2002; Kaartinen et al., 1995; Sanford et al., 1997). Interestingly, the *Tgfb2/3* double-knockout mouse showed severe midline closure defects, confirming the role of TGFβ signalling in VBW closure (Dünker and Krieglstein, 2002). Similarly, total knockout of different members of the homeobox gene family, the AP2α (TFAP2α) or aortic carboxypeptidase-like protein (ACLP, or AEBP1) transcription factors, and the Wnt signalling pathway cause different midline closure defects, including that of the VBW (Brewer and Williams, 2004; Layne et al., 2001; Snowball et al., 2015; Zhang et al., 1996, 2014). However, owing to the complete loss-of-function nature of all these models, it was impossible to identify specific cellular players in the closure process.

It has been shown that TGFβ signalling has distinct roles on specific target cells and tissues that are mediated by TGFβ receptors (Massagué, 2012). During embryogenesis, TGFβ superfamily ligands including decapentaplegic (Dpp), BMP and activin act as dose-dependent morphogens in a variety of fundamental embryonic processes such as left-right asymmetry and anterior-posterior patterning (Belenkaya et al., 2004; Entchev et al., 2000; Meno et al., 1996; Teleman and Cohen, 2000; Wu and Hill, 2009). Although all TGFβ morphogens signal via common receptors (TGFβR1/2 complex) their expression varies between tissues, explaining the differences in knockout mouse phenotypes. Furthermore, partial compensation may exist between TGFβ morphogens, leading to variable penetrance of the defect in individual morphogen knockout models. Cleft palate and defects in diverse midline components are evident in all individual TGFβ morphogen knockouts, suggesting their common involvement in midline closure (Kaartinen et al., 1995; Proetzel et al., 1995; Sanford et al., 1997). These analyses of the *Tgfb1/2/3* knockout models have provided invaluable insights into their role in embryonic development, but left open the question of the cell type(s) responding to their signals.

TGFβ signalling was shown to enhance cell motility by inducing reorganisation of the actin cytoskeleton (Boland et al., 1996; Edlund et al., 2002). TGFβ-induced transcriptional changes, mediated by SMAD2/3 transcription factors, control the actomyosin cytoskeleton by upregulating CITED1 and thereby promoting cell migration (Cantelli et al., 2015). TGFβ is also known to induce transgelin (*Tagln*) *in vitro* and *in vivo* (Adam et al., 2000; Hirschi et al., 1998; Yu et al., 2008) through SMAD binding to the *Tagln* promoter (Chen et al., 2003). TAGLN is an actin-binding cytoskeletal protein that is linked to increased cell motility and migration (Assinder et al., 2009; Elsafadi et al., 2016; Lin et al., 2009; Yu et al., 2008; Zhou et al., 2016).

Here we show that VBW closure relies on polarised migration of TAGLN^+^ myofibroblasts towards a TGFβ morphogen gradient originating from the epithelium of the primary body wall. The progeny of these embryonic myofibroblasts are maintained as a narrow line at the closed midline. Specific knockout of *Tgfbr2*, the receptor common to all TGFβ morphogens, from TAGLN^+^ cells leads to complete failure of VBW closure. By contrast, no such defects are evident when *Tgfbr2* is deleted from developing skeletal muscles. Our data reveal a principal role for myofibroblasts in mediating TGFβ signalling in VBW morphogenesis.

## RESULTS

### The ventral midline develops from convergent movement of TAGLN-expressing cells

**Fig. 1.**
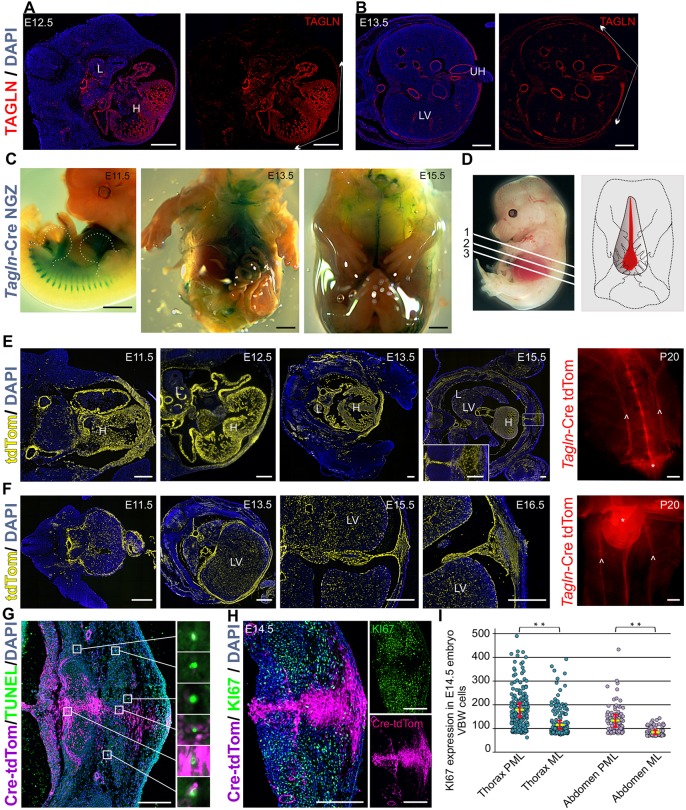
***Tagln*-Cre expression in the ventral midline and mitotic activity of TAGLN^+^ cells.** (A) Transverse section at a thoracic level in an E12.5 wild-type (WT) mouse embryo stained for TAGLN, showing expression in the primary VBW (area between arrows). (B) Transverse section at an abdominal level in an E13.5 WT embryo stained for TAGLN showing expression in the primary abdominal wall (area between arrows) that is encircling the umbilical hernia. (C) Whole-mount β-galactosidase staining in *Tagln*-Cre:Rosa26-NGZ at three embryonic stages. The expression of TAGLN is evident in the somite at E11.5 and localises to the midline area when VBW closure is complete. Dotted lines delineate forelimb and hindlimb. (D) (Left) Numbered lines indicate the level of transverse sections shown in (1) A,E,H, (2) B,F and (3) G. (Right) Schematic of midline (red) and para-midline (grey) areas presented in the KI67 analysis in H,I. (E,F) Expression of *Tagln*-Cre:Rosa26-tdTom in the thoracic (E) and abdominal (F) ventral midline over a 4 day time window during the closure process and at postnatal day (P) 20. TAGLN expression becomes restricted to the midline area with advanced gestation and this expression is maintained postnatally. Inset in E15.5 shows high magnification of the midline. Arrowheads indicate internal mammary/superior epigastric vessels and asterisk indicates the xiphisternum. (G) TUNEL assay for apoptosis in the ventral midline at E15.5. There is no obvious pattern of apoptosis in TAGLN^+^-derived cells in the midline. Boxes show examples of individual TUNEL^+^ cells in the midline and para-midline areas. (H) KI67 staining of the ventral midline at E14.5. Primary body wall remnant at this stage shows limited mitotic activity, which is evident in the KI67 channel. (I) Comparison of KI67 expression between midline (ML) primary VBW cells (tdTom^+^) and para-midline (PML) secondary body wall cells (tdTom^−^) in the thoracic and abdominal regions. Comparison was made on 200 cells from three different sections at each level; data presented as mean±s.e.m. **P<0.001, two-tailed *t*-test. H, heart; L, lungs; LV, liver; UH, umbilical hernia. Scale bars: 500 µm in A,B,F; 1000 µm in C and P20 in E,F; 200 µm in E,G,H.

We noticed high levels of TAGLN expression in the primary body wall area from early stages of VBW development (Fig. 1A,B). In order to follow the fate of TAGLN-expressing cells in primary body wall, we crossed the *Tagln*-Cre mouse strain to the Rosa26-NGZ [Gt(ROSA)26Sortm1(CAG-*lacZ*,-EGFP)Glh] reporter strain and performed whole-mount β-galactosidase staining at various embryonic stages. At E11.5 we detected *lacZ*-marked cells across the developing VBW and in the ventral aspect of the myotomes (Fig. 1C). Over the course of development the *lacZ*-labelled ventral area became progressively more restricted. At E13.5 the *lacZ*-labelled area adopts a triangular shape, wide at the caudal end. At E15.5, when the VBW has almost completely closed, only a narrow line of cells remains visible, besides the blood vessels that are also clearly labelled (Fig. 1C).

We also crossed *Tagln*-Cre to the Rosa26-tdTomato (tdTom) reporter mouse and analysed VBW closure in embryo sections (Fig. 1D). The widespread expression of the TAGLN reporter can be seen in the primary body wall at early embryonic stages (E11.5), but over a 4 day time window it became limited to a small area at the thoracic and abdominal midline (Fig. 1E,F), and the closure followed the craniocaudal axis similar to that seen in the *lacZ* whole-mount staining. Interestingly, the tdTom signal in the midline persisted into the juvenile postnatal growth phase and even into adulthood (Fig. S1A). This suggests that primary VBW cells are derived from TAGLN^+^ cells that continue to exist as resident cells in the midline of adult mice.

We next analysed whether the spatial narrowing of the tdTom^+^ cell population was due to cell death in the body wall or convergent migration of cells towards the midline. We found that the number of apoptotic cells in the ventral midline is low and we did not observe any difference in TUNEL labelling between tdTom^+^ and tdTom^−^ cells at E15.5 (Fig. 1G). The only apoptosis we observed was in the periphery of thoracic ribs at E15.5 and E16.5 where TAGLN^+^-derived (tdTom^+^) cells were also initially present, but eventually died (Fig. S1B,C). tdTom^+^ cells in the midline area showed much less mitotic activity than adjacent para-midline tdTom^−^ secondary body wall component cells, as revealed by KI67 staining (Fig. 1H). The average relative expression of nuclear KI67 signal in the midline area was 50% of that of the adjacent para-midline in the abdominal and thoracic regions (Fig. 1I). This excludes apoptosis as a mechanism behind the spatial narrowing of the tdTom signal and suggests that the secondary body wall of the ventral midline develops from proliferating lateral flank cells.

### TAGLN-expressing cells migrate towards the ventral midline

**Fig. 2.**
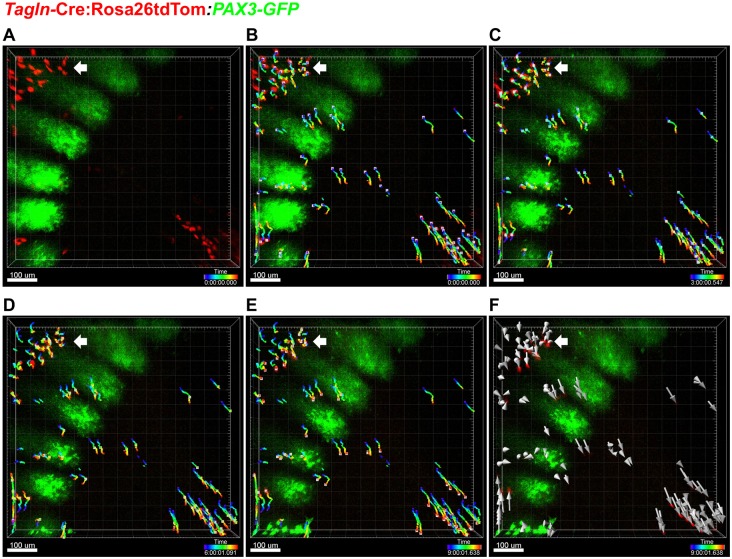
**Directional migration of TAGLN^+^ cells towards the ventral midline.** Still images from 9 h time-lapse (time shown bottom right) of *ex vivo* body wall explant culture. The VBW is located at the righthand side of each panel and dorsally located tdTom^+^ cells (white arrows) are in the left top corner. (A) Time zero, showing location of tdTom^+^ cells. (B) Time zero, with added tracks and migration paths. Each tdTom^+^ cell centre is labelled with a grey square and the path and time course of the journey are marked with a colour-coded line. (C) At 3 h VBW cells show directional migration towards the ventral midline, whereas dorsal cells show little change in position. (D,E) At 6 and 9 h, respectively, midline directional migration continues in VBW cells. (F) Trajectories and journey length in the analysed cells. Grey arrows indicate the direction and length of each migration path. VBW cells show consistent directional migration towards the midline, whereas dorsal cells show little change in position.

We next examined whether TAGLN-expressing cells of the primary VBW actively migrate from the somite region towards the midline. We used *ex vivo* body wall explants from E11.5 *Tagln-*Cre:Rosa26-tdTom:*PAX3-GFP* embryos (Fig. S2A). At this stage, GFP expression marks myoblasts in the somite and TAGLN-expressing cells of the VBW (tdTom^+^) are seen ventral to the somite (Fig. 2A). Over a 9 h period the tdTom^+^ cells at the flanks of the body wall showed active directional migration towards the midline (Fig. 2B-F, Movie 1) and the migrating cells exhibited cell protrusions and lamellipodia formation (Movie 2). By contrast, the more dorsally located tdTom^+^ resident cells (Fig. S2B) retained their existing localisation (arrow in Fig. 2A-F).

Using the *Tagln*-Cre:Rosa26-tdTom model, we performed topographical characterisation of the primary body wall at E14.5 and time-lapse confocal analysis of these cells in an *ex vivo* body wall explant culture. At the thoracoabdominal junction at E14.5 tdTom^+^ cells formed a cone-shaped mass that was wider caudally (Fig. S2C). Analysis over a 10 h time-lapse showed dynamic constriction of tdTom^+^ cells in a dorsoventral fashion, alongside the craniocaudal axis (Movie 3). This proves that TAGLN-expressing cells of the primary wall migrate from the somite region and continue to migrate towards the midline during VBW closure.

### TAGLN is downregulated when VBW closure is complete

**Fig. 3.**
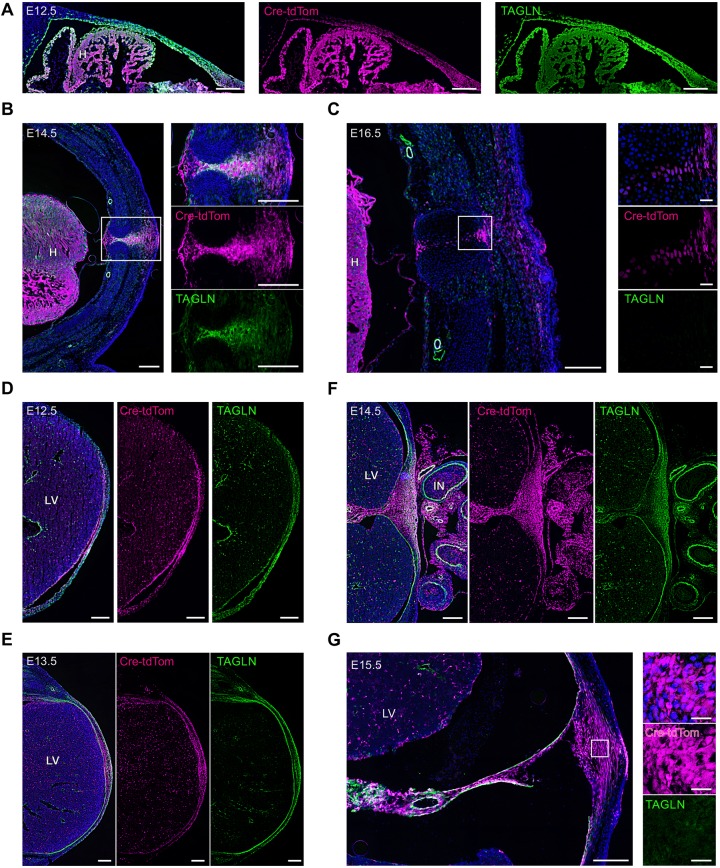
**TAGLN protein expression in the ventral midline during the closure process.** Transverse sections are shown of thoracic (A-C) and abdominal (D-G) VBW from *Tagln*-Cre:Rosa26-tdTom embryos stained with TAGLN antibody. (A) E12.5, showing the left primary body wall. Complete overlap between *Tagln*-Cre (tdTom) and TAGLN signals is seen. (B) At E14.5 there is still near complete overlap between the tdTom and TAGLN signals. A magnified view of the closing midline (boxed area in B) is shown to the right. (C) At E16.5 the thoracic midline has completely closed. The tdTom signal is still seen as a narrow line in the midline, but TAGLN signal cannot be identified. The magnified view of the midline (boxed area in C) shows the fine line of tdTom^+^ cells that have now become negative for TAGLN. (D) The abdominal ventral midline at E12.5, *Tagln*-Cre and TAGLN signals show complete overlap. (E) At E13.5 the *Tagln*-Cre-derived cells (tdTom^+^) of the primary body wall still express TAGLN. (F) At E14.5 the TAGLN signal area in the primary ventral midline is restricted compared with the tdTom signal area of the *Tagln*-Cre cells. (G) The ventral midline, labelled by tdTom, at E15.5 (same level as in F) has largely downregulated TAGLN. The magnified view of the midline (boxed area in G) shows tdTom^+^ cells of the midline that have now become negative for TAGLN. H, heart; LV, liver; IN, intestine. Scale bars: 200 µm, except 25 µm in higher magnification images in C,G.

In order to verify *Tagln*-Cre transgene specificity and distinguish between TAGLN-expressing cells and their progeny we analysed the expression of native TAGLN protein. In the early stages of VBW closure (E12.5-E13.5) we found complete overlap between *Tagln*-Cre:Rosa26-tdTom expression and TAGLN antibody signal in the primary body wall in both the thoracic and abdominal regions (Fig. 3A,D,E). This was still the case until E14.5, when nearly full overlap was noted between the two signals in the primary ventral wall (Fig. 3B,F). However, from E15.5 onwards, TAGLN signal in the closed midline diminished in the thoracic (Fig. 3C) and abdominal (Fig. 3G) midline regions and, using confocal microscopy, we noticed that TAGLN was no longer expressed in the residual *Tagln-*Cre area (Fig. 3C,G, right). This indicates that mature midline cells, which are progeny of TAGLN^+^ cells, progressively switch off *Tagln* gene expression when their migration is complete and the ventral midline has fused.

### TAGLN-expressing myofibroblasts drive midline closure

**Fig. 4.**
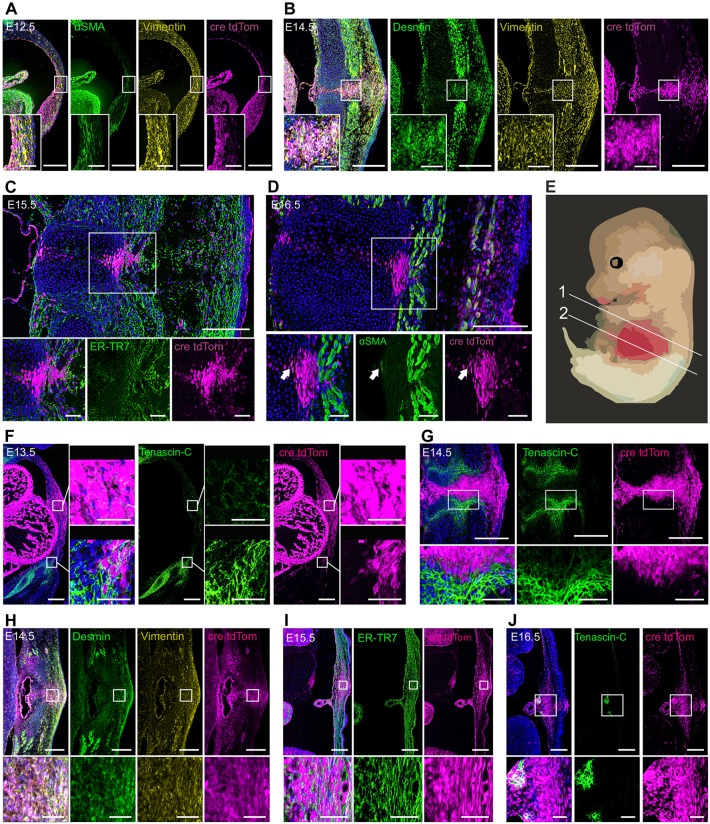
**Characterisation of ventral midline cells in *Tagln*-Cre:Rosa26-tdTom during VBW closure.** Expression of smooth muscle contractile proteins (A-D,H) in the primary wall is more evident at early stages of midline closure. (A) αSMA and vimentin are expressed in the thoracic primary body wall at E12.5 and correlate with tdTom signal. Insets are magnified views (at cellular level) of the boxed areas. (B) At E14.5 primary body wall cells labelled by tdTom are still strongly positive for vimentin and express the smooth muscle intermediate filament protein desmin. (C) E15.5 midline cells are immunopositive for the fibroblast marker ER-TR7. Inset shows the ventral midline area (boxed) at higher magnification. (D) When the thoracic midline is fully closed at E16.5 the residual primary midline cells still labelled by tdTom have now downregulated αSMA. As shown in the higher magnification inset, only a small number of cells (arrow) of the midline show expression of αSMA. (E) Numbered lines indicate the level of transverse sections shown in (1) A-D,F,G and (2) H-J. (F,G) Tendon markers are absent in the primary body wall. (F) Tendon marker tenascin-C is expressed at E13.5 around the rib primordium and just lateral to primary elements (bottom box), and sporadic low-level expression is seen in the primary body wall (top box). (G) At E14.5 no tenascin-C expression is seen in the primary body wall in the midline. Sternal primordium cells express tenascin-C and are seen encircling the primary body wall cells. (H-J) Abdominal primary body wall is made of myofibroblasts. (H) In the abdominal midline at E14.5, primary body wall cells express vimentin and desmin. (I) At E15.5 the cells of the abdominal midline are immunopositive for the fibroblast marker ER-TR7. (J) At E16.5 the ventral midline has fully closed and resident tdTom^+^ cells are seen in the midline. Tenascin-C expression can be detected in the edges of the falciform ligament, but not at the midline. Scale bars: 100 µm.

We further characterised the ventral midline cells during the closure process. The TAGLN-expressing cells of the ventral midline express the mesodermal and fibroblast marker vimentin throughout the studied time points (Fig. 4A,B,H). The expression of smooth muscle cell markers, however, was gestation dependent. In the thorax, smooth muscle alpha actin (αSMA) was expressed more abundantly at early time points (Fig. 4A), whereas when the ventral midline was fully closed by E16.5, αSMA expression was more limited to a small number of midline cells ventral to the closed sternum (Fig. 4D). Similarly, early in the closure process desmin is expressed in the TAGLN^+^ VBW cells in both the thoracic and abdominal areas (Fig. 4B,H). These data suggest that at the time of midline closure the migrating cells express several contractile and cytoskeletal proteins (αSMA, TAGLN, vimentin and desmin), some of which are downregulated in the mature midline.

The TAGLN^+^ cells of the midline were also immunopositive with the ER-TR7 (anti-reticular fibroblast and reticular fibre antibody 7) monoclonal antibody, confirming their fibroblast-like nature. The ER-TR7 signal was evident at late time points in the closure process in both the thoracic and abdominal areas (Fig. 4C,I). Of note, the cells of the midline did not express tendon cell markers. Tenascin (an extracellular matrix glycoprotein) expression at E13.5 and E14.5 was strong just lateral to the primary VBW, and cells expressing tenascin-C appeared to be encapsulating the TAGLN^+^-derived midline cells (Fig. 4F,G). Moreover, when the ventral closure process was complete, at E16.5, we did not detect tenascin-C expression in the resident TAGLN^+^ cells in the midline (Fig. 4J).

### TGFβ epithelial-mesenchymal signalling in the primary VBW

**Fig. 5.**
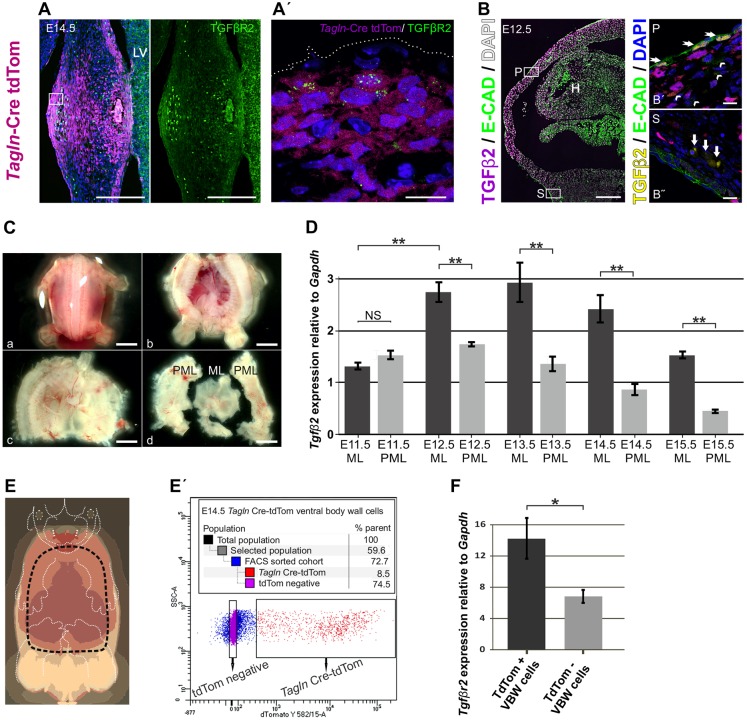
**TGFβ2 and TGFβR2 in the VBW.** (A) Transverse section in the abdominal VBW at E14.5 showing expression of TGFβR2 focused in the primary body wall area (labelled by tdTom) in the ventral midline. (A′) Confocal image of the boxed area in A, showing high-level TGFβR2 expression in tdTom^+^ cells beneath the epithelium. (B) Transverse section in the mid thoracic area at E12.5 *Tagln*-Cre:Rosa26-tdTom mouse embryo stained for TGFβ2 and E-cadherin to label epithelium. TGFβ2 protein is abundant in the midline area of the primary body wall (tdTom channel is removed to expose the TGFβ2 signal). (B′) Confocal image of the primary body wall area (box P) showing strong TGFβ2 expression in the epithelium (arrows) and weaker signalling in the subdermal layer (arrowheads). (B″) Confocal image of the secondary body wall area (box S) showing weak TGFβ2 signal in the subdermal layer (arrows). (C) Midline (ML) and para-midline (PML) ventral wall dissection in an E12.5 WT mouse embryo. (Ca) The embryo was decapitated and the tail excised. (Cb) The dorsal body wall was opened para-sagittal and the thoracic and abdominal organs were exposed. (Cc) The embryo was eviscerated, taking care to preserve the thin primary body wall. (Cd) The thin primary (midline) body wall was carefully dissected from the secondary (para-midline body) wall and sufficient margins were removed from both segments to avoid transitional areas. (D) RT-qPCR comparing *Tgfb2* expression in the midline and para-midline of WT mouse embryos between E11.5 and E15.5. There is an anatomical and temporal *Tgfb2* gradient in the midline during the closure period. Error bars are s.e.m.; each time point presented is from at least three biological replicates each containing tissue from at least five embryos. (E) Schematic of E14.5 embryo. The VBW delineated by the dashed line was dissected from *Tagln*-Cre:Rosa26-tdTom embryos and FACS sorted for tdTom signal. (E′) The FACS-sorted cohort. tdTom^+^ cells only accounted for an average of 15% of the total cell population of the VBW (as shown in E). (F) RT-qPCR on the FACS-sorted cells showed higher expression of *Tgfbr2* in tdTom^+^ ventral midline cells. Error bars indicate s.e.m.; data presented are from three biological replicates each containing cells from tissue derived from at least seven embryos. **P<0.001; *P<0.05; NS, non-significant; two tailed *t*-test. H, heart; LV, liver; P, primary body wall; S, secondary body wall. Each experiment shown in D, E and F was repeated at least three times. Scale bars: 200 µm, except 10 µm in A′,B′,B″.

We found high levels of TGFβR2 expression in the ventral midline at E13.5 and 14.5 that localises to the primary body wall area labelled with tdTom (Fig. 5A). Using confocal microscopy we noted that TGFβR2 is abundant in the primary VBW on tdTom^+^ cells just beneath the epithelial layer (Fig. 5A′). In a similar fashion, TGFβ2 protein expression is profuse in the primary VBW from E12.5, as compared with the secondary body wall area (Fig. 5B). This TGFβ2 signal is particularly strong in the epithelium and to a lower degree in the subdermal layer, as shown by confocal microscopy (Fig. 5B′). We detected far less TGFβ2 in the secondary body wall, where it was restricted primarily to the subdermal layer (Fig. 5B″).

In order to prove the presence of a TGFβ2 gradient in the VBW during closure, we dissected the midline and para-midline VBWs from wild-type mouse embryos (as shown in Fig. 5C) at different time points and tested the expression of *Tgfb2* by RT-qPCR. We found an anatomical and temporal *Tgfb2* gradient towards the midline during VBW closure time points. *Tgfb2* RNA expression in the midline dramatically increased at E12.5, peaked at E13.5 and then started to tail off at E15.5 when the VBW is almost fully closed (Fig. 5D).

Furthermore, to confirm the high levels of TGFβR2 expression in TAGLN^+^ cells we dissected the VBW (as shown in Fig. 5E) from *Tagln*-Cre:Rosa26-tdTom embryos, isolated tdTom^+^ and tdTom^−^ cells by FACS and tested *Tgfbr2* expression by RT-qPCR. Interestingly, tdTom^+^ cells only accounted for an average of ∼15% (from all experiments) of the total ventral midline cell population at E14.5 (Fig. 5E′). Yet, *Tgfbr2* expression is significantly higher in *Tagln-*Cre:tdTom^+^ than in tdTom^−^ VBW cells (Fig. 5F).

### TGFβ signalling in TAGLN^+^ cells is required for midline closure

**Fig. 6.**
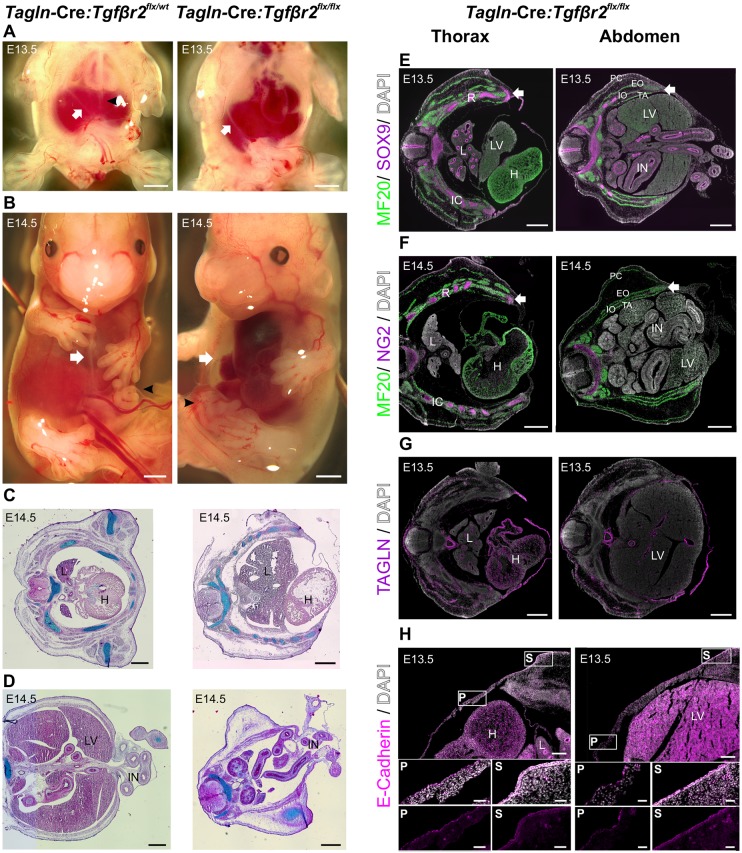
***Tagln-*Cre:*Tgfbr2^flx/flx^* embryos develop VBW closure defects.** (A,B) Morphological comparison between *Tagln-*Cre:*Tgfbr2^flx/flx^* and *Tagln-*Cre:*Tgfbr2^flx/wt^* mouse embryos. (A) E13.5 *Tagln-*Cre:*Tgfbr2^flx/flx^* embryos show a translucent ventral midline, a more lateral limit to the secondary body wall (arrow) and absence of midline raphe (arrowhead) when compared with *Tagln*-Cre:*Tgfbr2^flx/wt^*. (B) The ventral midline closure defect in *Tagln-*Cre:*Tgfbr2^flx/flx^*. A thin membrane covers the VBW cavities, as compared with the nearly closed thoracic midline in the WT (arrow) and the embryos show a large exomphalos compared with the physiological umbilical hernia in the WT (arrowhead). (C) Transverse section in mid-thorax at E14.5 in WT (left) and *Tagln*-Cre:*Tgfbr2^flx/flx^* (right), with Alcian Blue staining to delineate ribs and counterstaining with Nuclear Fast Red. The VBW is composed of a thin sac in the mutant, whereas the two lateral sternebrae are nearly meeting in the midline in the WT. (D) Transverse section at level of the umbilical hernia at E14.5 in WT (left) and *Tagln-*Cre:*Tgfbr2^flx/flx^* (right), with Alcian Blue staining to delineate ribs and counterstaining with Nuclear Fast Red. In the WT only a small physiological umbilical hernia is present and the small intestine is returning to the abdominal cavity, whereas the mutant shows a large exomphalos defect and very few bowel loops are present in the abdominal cavity. (E-H) Characterisation of cell type in *Tagln-*Cre:*Tgfbr2^flx/flx^* thoracic (right) and abdominal (left) body wall by immunohistochemistry. (E) E13.5 mutant embryos show normal lateral body wall muscles (MF20^+^) and ribs (SOX9^+^), whereas the ventral midline is made of a thin sac. Condensations of SOX9^+^ and MF20^+^ cells (arrow) are seen just lateral to the VBW in the thoracic and abdominal areas, respectively. (F) E14.5 mutant embryo shows very little progression in secondary element migration, and the condensation of chondrocyte and myocyte (arrow) is still seen lateral to the VBW in the thoracic and abdominal compartments, respectively. (G) The VBW of *Tagln-*Cre:*Tgfbr2^flx/flx^* still expresses TAGLN. (H) The skin covering the premature VBW in *Tagln-*Cre:*Tgfbr2^flx/flx^* is made of a single layer of squamous epithelial cells (insets P), while in the secondary elements multilayered cuboid epithelium covers the lateral body wall (insets S). Bottom row of insets shows E-cadherin channel. H, heart; L, lungs; LV, liver; IN, intestine; P, primary body wall; S, secondary body wall; TA, transverses abdominis; IO, internal oblique; EO, external oblique; PC, panniculus carnosus; IC, intercostal muscles; R, rib. Scale bars: 1000 µm in A,B; 500 µm in C-G; 200 µm in H, 50 µm in insets.

TGFβ signalling plays an important role in midline closure (Dünker and Krieglstein, 2002). However, the specific cell types that mediate the response to TGFβ remain largely unknown. We knocked out *Tgfbr2* selectively in TAGLN^+^ cells and observed a dramatic VBW closure defect in 100% of mutant mice (*n*=10). In the *Tagln-*Cre:*Tgfbr2^flx/flx^* embryos, a thin primary body wall covers the ventral midline and secondary body wall elements fail to migrate towards the midline after E13.5 (Fig. 6A). At E14.5 the thoracic and abdominal cavities are only covered with a thin and membranous primary body wall (Fig. 6B) that is less than one-third the thickness of the primary body wall of the wild type.

We characterised this mutant model by serial sectioning of the thoracic and abdominal regions. Secondary body wall components failed to migrate beyond two-thirds of the lateral wall. This failure of migration was most evident at E14.5; in the thoracic region the ribs are malaligned, fail to progress ventrally and the sternal primordium is completely absent. By contrast, wild-type embryos showed formed sternebrae separated by a narrow residual primary body wall in the midline (Fig. 6C). Similarly, in the abdominal region of the mutant we detected large herniation of the abdominal contents at E14.5. By contrast, the wild-type embryo showed only a small physiological umbilical hernia at this stage and the small intestine had largely returned to the abdominal cavity (Fig. 6D). All *Tagln-*Cre:*Tgfbr2^flx/wt^* (*n*=20) control embryos showed normal developmental milestones similar to those of the wild type and did not exhibit any VBW closure defects (Fig. S3A).

Using immunohistochemistry, we showed that there is little progression of the lateral ribs and muscles in *Tagln-*Cre:*Tgfbr2^flx/flx^* mice. At E13.5, we noted a condensation of chondrogenic cells (SOX9^+^) and skeletal muscle cells (expressing sarcomeric myosin) at the most rostral part of the secondary body wall in the thoracic and abdominal regions, respectively (Fig. 6E). We detected a similar pattern of SOX9 and myosin expression at E14.5, with very limited progression of secondary elements having taken place during the extra gestational day (Fig. 6F). We propose that in *Tagln-*Cre:*Tgfbr2^flx/flx^* mice, secondary wall component cells have lost the signalling required for their patterning and hence condense at the junction between the primary and secondary body walls.

The immature primary body wall is made of epithelium and a thin layer of mesodermal cells expressing vimentin (Fig. S3B). Still, this thin membrane maintains TAGLN expression (Fig. 6G) and preserves its TGFβ2 secretory activity (Fig. S3D). Epithelial cover of the VBW appears to be preserved in the mutant and resembles that of the wild type. In *Tagln-*Cre:*Tgfbr2^flx/flx^* embryos an immature single layer of squamous epithelium covers the thin primary ventral wall, while multilayered cuboid epithelial cells cover the secondary body wall in the flanks (Fig. 6H). Similar patterning of epithelial cells is seen in the wild-type embryo (Fig. S3C). In the knockout model, TGFβR2 can be detected in the secondary element areas laterally, but TGFβR2 is scarce in the primary areas in the thoracic (Fig. S3E) and abdominal (Fig. S3F) regions. This further confirms the selective elimination of TGFβR2 in our knockout model and indicates that a minority subset of cells in the primary body wall drives ventral midline closure through TGFβ signalling. Furthermore, this strongly suggests that different mechanisms and cell types are involved in the closure of the lateral and ventral body walls. The secondary body wall elements in the dorsal half of the body wall were anatomically normal, with distinct layers of intercostal muscles and all four lateral abdominal wall muscle layers (Fig. 6C-F). Of note, the more caudal VBW of the pelvic region closed normally and we did not observe any bladder exstrophy (Fig. S3G). The knockout model exhibited severe cardiac and major vessel defects, as have often been observed in other cases of midline closure defects. The aortic arch showed aneurysmal changes (Fig. 6B) and cardiac septal defects were also evident (Fig. 6F). Nevertheless, all mutant embryos collected until E14.5 were viable and showed active cardiac function (Movie 4). These defects most likely contributed to the late embryonic lethality. The embryos did not survive beyond E15.5 and showed an obvious VBW defect and intraembryonic bleeding (Fig. S3H).

### TGFβ signalling in myogenic and chondrogenic progenitors is not essential for VBW closure

**Fig. 7.**
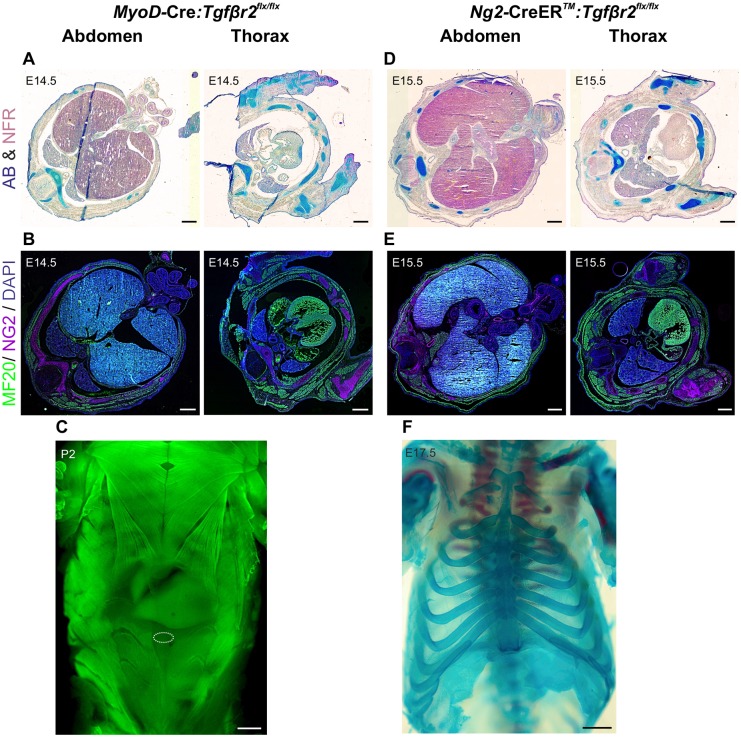
***Tgfbr2* knockout in myogenic and chondrogenic cells does not affect midline closure.** (A,B) Transverse sections in the thoracic and abdominal region of an E14.5 *MyoD*-Cre:*Tgfbr2^flx/flx^* embryo. (A) Alcian Blue (AB) and Nuclear Fast Red (NFR) staining show normal developmental milestones, comparable to the WT (see Fig. 6C,D). (B) Normal muscle (MF20^+^) and chondrocyte [NG2 (CSPG4)^+^] development in the midline area of the mutant mouse. (C) Whole-mount MF20 staining of a 2-day-old pup, showing normal muscle development in the midline postnatally in the mutant. The umbilicus site is marked with a dotted circle. (D,E) Transverse sections in the thoracic and abdominal region of an E15.5 *NG2-*CreER™:*Tgfbr2^flx/flx^* embryo. (D) Alcian Blue and Nuclear Fast Red staining show normal developmental milestones, comparable to WT. (E) Normal muscle (MF20^+^) and chondrocyte (NG2^+^) development in the midline area of the mutant mouse. (F) Whole-mount Alizarin Red and Alcian Blue showing normal rib cage development and fused sternum in the midline at the fetal stage in the mutant. Scale bars: 500 µm in A,B,D,E; 1000 µm in C,F.

We next analysed whether, in addition to myofibroblasts, the main components of the body wall – myoblasts and chondrocytes – respond to the TGFβ gradient during midline closure. *Tagln* is also expressed in embryonic myoblasts (Li et al., 1996), raising the possibility that the midline defect might arise due to TGFβR2 targeting in myogenic cells. To rule this out we crossed the *Tgfbr2^flx/flx^* strain to the *MyoD-*Cre mouse line (Chen et al., 2005). MyoD (MYOD1) is abundantly expressed in myoblasts before and at the time of midline closure in intercostal and abdominal wall myoblasts (Chen et al., 2001). Importantly, we observed no midline closure defects in the *MyoD-*Cre:*Tgfbr2^flx/flx^* embryos (Fig. 7A,B) and their body wall musculature was indistinguishable from that of wild-type mice (Fig. 6C,D). Postnatally, these mice had a fully closed ventral midline and normal ventral skeletal muscles (Fig. 7C).

To assess the importance of TGFβ signalling in cartilage primordium we used the *NG2-*CreER^TM^ mouse line (Zhu et al., 2011) crossed to the *Tgfbr2^flx/flx^* strain and administered tamoxifen at E12.5. Similarly, *NG2-*CreER^TM^:*Tgfbr2^flx/flx^* embryos did not exhibit any defects in midline closure (Fig. 7D,E) and the fetuses had a normal rib cage and fused sternum (Fig. 7F). We can thus conclude that TGFβ signalling in myoblasts and chondrocytes is not necessary for VBW formation.

## DISCUSSION

We describe a population of cells in the primary VBW that orchestrates the closure of the ventral midline in mice. These pioneering myofibroblasts respond to a spatiotemporal TGFβ gradient originating from the primary body wall and migrate towards the midline. We show that specific ablation of TGFβ receptor in myofibroblasts but not in myoblasts or chondrogenic cells leads to a severe midline closure defect.

*Tagln* is expressed in a variety of developing tissues and is not a specific smooth muscle marker during embryogenesis. It is expressed in the myotome as early as E9.5 and maintained until nearly E11.5 (Li et al., 1996). At E11.5 we detected *Tagln* expression in the hypaxial myotome region that extends to cover the primary VBW. Later on in embryogenesis, *Tagln* expression is confined to a narrower area in the primary body wall, in addition to being expressed in the blood vessels and gut wall. Previous *in situ* hybridisation studies at E14.5 show *Tagln* expression confined within the primary midline mesenchyme in a similar pattern to our observations (Diez-Roux et al., 2011). These data imply that the primary body wall originates from the convergent migration of lateral somitic TAGLN^+^ mesodermal cells. In line with the migratory status of the TAGLN^+^ myofibroblasts, these cells express a number of cytoskeletal regulators that are normally found in migratory cells. This is also supported by recent studies that showed that TAGLN expression enhances the migration of metastatic cells (Elsafadi et al., 2016; Yu et al., 2008; Zhou et al., 2016).

TGFβ is a known regulator of midline closure (Dünker and Krieglstein, 2002) and its localised signalling gradient is important in the development of a number of embryonic tissues (Massagué, 2012). *Tgfb2* RNA is expressed in the somites from E9.5 and is later confined to the dermatome and VBW at E12.5 (Dickson et al., 1993). At later stages, *Tgfb2* is expressed in the primary midline at E14.5, but not in the para-midline regions (Diez-Roux et al., 2011). We show a TGFβ2 gradient in the VBW during the closure process. The expression of *Tgfb2* peaks at E13.5 when the majority of secondary element patterning occurs. Importantly, we detected the highest expression of the ligand in epithelial cells, suggesting a paracrine effect in recruiting somite-derived TAGLN^+^ TGFβR2^+^ cells to the ventral midline. A similar mechanism involving the TGFβ superfamily and cell motility was demonstrated in dorsal closure in the *Drosophila* embryo. Jun N-terminal kinase (JNK)-activated Dpp was shown to rearrange the cytoskeleton and generate morphogenetic cell changes leading to dorsal closure (Hou et al., 1997; Sluss and Davis, 1997).

*Tgfbr2* knockout in *Tagln*-expressing cells leads to a thinner primary body wall and subsequent complete failure of midline closure. However, this thin primary body wall in the *Tagln-*Cre:*Tgfbr2^flx/flx^* is made of ectodermal and mesodermal components and preserves TAGLN and TGFβ2 expression. By contrast, the phenotype and cellular components in other VBW closure defects, such as thoracoabdominoschesis, are anatomically different and sometimes lack any tissue cover to the endoderm.

Considering the various complexities and severities of VBW closure defects and the effects of different genetic mutations in generating them we propose a model of successive cell movements during VBW closure. In the first cell migration wave at E9, following embryo turning, mesodermal cells originating from the dermatome and epithelial cells provide the first tissue cover to the embryonic endoderm. These early cells secrete TGFβ and initiate a second wave of migration of myofibroblasts expressing TAGLN and TGFβ receptors. In the third wave of migration, the secondary body wall elements, including progenitors of skeletal muscles and ribs, develop.

We provide several lines of evidence to support active migration of these myofibroblasts towards the midline: we demonstrate their directed migration in an *ex vivo* culture model; show their elevated expression of cytoskeletal components (TAGLN, αSMA and desmin); and reveal their high-level expression of TGFβR2, making them responsive to the morphogen gradient. We propose that the pre-patterning of the body wall by myofibroblasts is required for the final cellular wave in body wall development, in which skeletal and myogenic progenitors reach their correct anatomical location. Indeed, elimination of TGFβR2 in myofibroblasts results in failed VBW closure, encompassing both skeletal and myogenic components. By contrast, *Tgfbr2* knockout in chondrogenic or myogenic cells does not result in this defect.

Our model of VBW closure might provide a logical explanation for the different anatomical configurations seen in different VBW defects. In thoracoabdominoschesis, there is complete absence of any ventral tissue cover, whereas in our and other models of exomphalos a thin ‘sac’ covers the ventral midline (Brewer and Williams, 2004; Carnaghan et al., 2013; Dünker and Krieglstein, 2002; Eng et al., 2012). The anatomy of the defect might reflect the stage of cellular wave failure. The first cell wave is probably not TGFβ dependent, but rather linked to epithelial factors. Knockout of pathways involved in epithelial patterning (AP2α, Wls or β-catenin) leads to thoracoabdominoschesis defects in which the ventral body cavities lack any cover and are directly exposed to the amniotic fluid (Brewer and Williams, 2004; Zhang et al., 1996, 2014). In exomphalos, initial tissue cover is present and the defect results from failure of advancement of later cell waves. At the other end of the spectrum, milder defects may be due to failure of the third cell wave. In prune belly syndrome there is lack of skeletal muscle cover to the ventral midline and a thicker sac covers the abdominal cavity (Jennings, 2000). This indicates that several, temporally regulated mechanisms drive ventral midline closure and the type of the VBW defect depends on the stage at which the insult takes place.

Collectively, we demonstrate here that ventral midline closure relies on a dynamic TGFβ-dependent recruitment of myofibroblasts. Our data suggest a model whereby sequential waves of cellular movements occur in VBW development that may explain the diversity of phenotypes in VBW closure defects.

## MATERIALS AND METHODS

### Animals

Mice were housed and bred in the University of Manchester animal facility. Mouse models have been published previously: *Tagln-*Cre (Li et al., 1996), *Pax3^GFP^* (Relaix et al., 2005), *MyoD-*Cre (Chen et al., 2005) and *NG2-*CreER^TM^ (Zhu et al., 2011). *Tagln*-Cre mice were crossed with Rosa26 tdTom (Madisen et al., 2010) and CD1 Rosa NGZ/*lacZ* (Soriano, 1999) reporter mice. *Tagln-*Cre:*Tgfbr2^flx/flx^*, *MyoD-*Cre:*Tgfbr2^flx/flx^* and *NG2-*CreER^TM^:*Tgfbr2^flx/flx^* were obtained by crossing *Tagln-*Cre, *MyoD-*Cre and *NG2-*CreER^TM^, respectively, to *Tgfbr2^flx/wt^* mice (Chytil et al., 2002) and the offspring were crossbred to obtain homozygous embryos, as confirmed by genotyping. Recombination in *NG2-*CreER™:*Tgfbr2^flx/flx^* was triggered at E12.5 by a single intraperitoneal dose of tamoxifen and progesterone (1 mg and 0.5 mg per 10 g body weight, respectively). All animal work was conducted in accordance with UK Home Office regulations and was approved under license no. 707435.

### Immunofluorescence staining

Embryos were fixed in 4% paraformaldehyde (PFA) in PBS for 2-4 h (depending on their gestational stage) and dehydrated in a sucrose gradient overnight. Embryos were embedded in an OCT (Clinipath) mould and snap-frozen in liquid nitrogen. 10 μm cryosections were cut with a CM3050 cryostat (Leica). Slides were washed in PBS, PBS with 0.2% Tween 20 (three times for 5 min each) and then blocked in incubation buffer (10% normal donkey serum, 1% BSA, 0.2% Tween 20 in PBS) for 4 h. Slides were incubated with primary antibody overnight at 4°C. Slides were then washed with PBS containing 0.2% Tween 20 and blocked in a second incubation buffer (1% BSA and 0.2% Tween 20 in PBS) for 1 h at room temperature before adding fluorescently labelled secondary antibodies (1:500; Life Technologies). Slides were incubated for 1 h at room temperature with secondary antibodies specific to the primary antibody host species. Slides were washed as described above and mounted in Vectashield mounting medium with DAPI (Vector Labs). Slides were imaged using a Zeiss Axio Imager M2. Zeiss Zen software was used for image analysis (tiling, counting). Whole-mount immunohistochemistry is described elsewhere (Merrell et al., 2015). Antibodies listed in Table S1.

### Whole-mount β-galactosidase staining

Fixed embryos (as above) were permeabilised in 1% Triton X-100 and 0.4% NP40 in PBS for 4 h and then incubated overnight for β-galactosidase activity at 37°C as described (Tajbakhsh et al., 1994). Embryos were imaged using a Zeiss Axio Zoom microscope and Zeiss Zen software was used for image analysis.

### Alcian Blue and Nuclear Fast Red staining

Cryosection slides (as above) were washed in PBS, PBS with 0.2% Tween 20 (three times for 5 min each) and 3% acetic acid (three times for 5 min each). Alcian Blue (Sigma-Aldrich, 1% solution in 3% acetic acid) was then added for 20 min. The slides were then washed in 3% acetic acid followed by PBS (5 min each) and counterstained with Nuclear Fast Red (Sigma-Aldrich, 0.1% in 5% aluminium sulphate) for 5 min.

### TUNEL staining for apoptosis

Cryosection slides (as above) underwent antigen retrieval in 1 M sodium citrate (pH 6) by boiling for 45 s. Cooled slides were then washed in PBS (three times for 5 min each) and peroxidase block (3% H_2_O_2_ in PBS) for 10 min. TUNEL assay (In Situ Cell Death Fluorescein, Roche) was performed as per the manufacturer's protocol.

### Whole-mount Alcian Blue and Alizarin Red staining

The protocol is described by McLeod (1980). Briefly, embryos were fixed in 95% ethanol for 24 h, placed in acetone for 24 h and then in the staining solution for 24 h at 37°C. The tissue was then cleared in 1% KOH in 20% glycerine for 1 week and stored in pure glycerine. Alcian Blue (lot MKBV1360V) and Alizarin Red (lot MKBS9114V) were obtained from Sigma-Aldrich.

### Cell proliferation

Cell proliferation of VBW cells was assessed in E14.5 embryo sections using KI67 antibody (Table S1). Comparison was made between *Tagln*-Cre:Rosa26-tdTom midline cells and TAGLN^−^ para-midline cells in the abdominal and thoracic regions as shown in Fig. 1B. Nuclei of tdTom^+^ and tdTom^−^ cells were marked in the Zen software in each image field at 20× (without KI67 staining overlay). A minimum of 100 cells from each group per section were labelled and the relative KI67 signal intensity (calculated in the Zen software) was exported into Excel (Microsoft) for statistical analysis.

### Tissue dissociation, fluorescence-activated cell sorting (FACS) and RNA extraction

In the *Tgfb2* gradient experiment, the VBW area from wild-type embryos was dissected as described in Fig. 4C and the midline and para-midline tissue samples were placed on dry ice during dissection. Once tissue from at least five embryos had been collected, Trizol (Life Technologies) was added and RNA extraction was conducted according to the kit protocol. In the *Tgfbr2* expression experiment, the VBW area from *Tagln*-Cre:Rosa26-tdTom embryos was dissected as described in Fig. 4E. The tissue was incubated in Krebs-Ringer-Hepes (KHR) containing 2 mg/ml collagenase type 2 (Worthington), 50 µg/ml DNase 1, 2.5 mM glucose and 5% fetal bovine serum (FBS) at 37°C in a shaking water bath. Dissociation cycles of 30 min continued until the tissue was digested to a homogenous cell suspension. Cells were diluted (1:5) in ice-cold Hank's Balanced Salt Solution (HBSS) with 20% FBS, centrifuged (600 ***g***, 5 min), resuspended in HBSS with 2% FBS and passed through a 50 µm cell strainer. Cells were then sorted using a BD Biosciences FACSAria for tdTom signal into tdTom^+^ and tdTom^−^ groups. Cells were collected directly into Trizol LS (Life Technologies) and RNA extraction was conducted according to the kit protocol.

### RT-qPCR

Extracted RNA (as above) was resuspended and quantified with a NanoDrop 2000 (ThermoFisher). cDNA was synthesized using the Superscript IV Kit (ThermoFisher) and 20 ng of cDNA was used for PCR amplification. Primers (forward and reverse) were: *Tgfbr2*, 5′-GAACGACAAGAACATTACTCTGGAG-3′ and 5′-GATGTCCTTCTCTGTTTTCCACGA-3′; *Tgfb2*, 5′-TCGACATGGATCAGTTTATGCG-3′ and 5′-CCCTGGTACTGTTGTAGATGGA-3′. A Roche LightCycler 96 and FastStart Essential DNA Green Master Mix were used for qPCR experiments, which were performed in biological and technical triplicate. Absolute quantification of each target was performed using a standard curve as a reference in Roche LightCycler software version 1.5.

### Time-lapse confocal laser scanning microscopy

E11.5-E14.5 *Tagln*-Cre:Rosa26-tdTom or *Tagln-*Cre:Rosa26-tdTom:*PAX3*-GFP embryos were decapitated and the trunk full circumference was maintained. The body wall explant was placed on a glass-bottom 35 mm tissue culture disc (WPI, FD3510-100), stabilised with Phenol Red-free growth factor-reduced Matrigel (Corning, 356231), then standard cell culture medium (DMEM with 10% FBS) was added and then cultured for 6-16 h. The explants were imaged in a laser scanning confocal microscopy system (Leica SP8 inverted gSTED) maintained at 37°C and 5% CO_2_ with a humidiﬁer. *z*-slices were acquired using a ×10 objective every 10-20 min. Four-dimensional datasets were analysed with Leica confocal and Imaris (Bitplane) software.

### Statistics

Quantitative data are presented as mean and s.e. Two-tailed Student's *t*-test was used for statistical analysis and *P*<0.05 was considered significant.
